# Contrasting the Genetic Patterns of Microbial Communities in Soda Lakes with and without Cyanobacterial Bloom

**DOI:** 10.3389/fmicb.2018.00244

**Published:** 2018-02-22

**Authors:** Ana P. D. Andreote, Francisco Dini-Andreote, Janaina Rigonato, Gabriela Silva Machineski, Bruno C. E. Souza, Laurent Barbiero, Ary T. Rezende-Filho, Marli F. Fiore

**Affiliations:** ^1^Center for Nuclear Energy in Agriculture, University of São Paulo, Piracicaba, Brazil; ^2^Microbial Ecology Cluster, Genomics Research in Ecology and Evolution in Nature, Groningen Institute for Evolutionary Life Sciences, University of Groningen, Groningen, Netherlands; ^3^Observatoire Midi-Pyrénées, Géosciences Environnement Toulouse, Institut de Recherche pour le Développement, Centre National de la Recherche Scientifique, Université Paul Sabatier, Toulouse, France; ^4^Faculty of Engineering, Architecture and Urbanism and Geography, Federal University of Mato Grosso do Sul, Campo Grande, Brazil

**Keywords:** arsenic, alkaline lakes, metagenomic, nitrogen, saline lakes, sulfur

## Abstract

Soda lakes have high levels of sodium carbonates and are characterized by salinity and elevated pH. These ecosystems are found across Africa, Europe, Asia, Australia, North, Central, and South America. Particularly in Brazil, the Pantanal region has a series of hundreds of shallow soda lakes (ca. 600) potentially colonized by a diverse haloalkaliphilic microbial community. Biological information of these systems is still elusive, in particular data on the description of the main taxa involved in the biogeochemical cycling of life-important elements. Here, we used metagenomic sequencing to contrast the composition and functional patterns of the microbial communities of two distinct soda lakes from the sub-region Nhecolândia, state of Mato Grosso do Sul, Brazil. These two lakes differ by permanent cyanobacterial blooms (Salina Verde, green-water lake) and by no record of cyanobacterial blooms (Salina Preta, black-water lake). The dominant bacterial species in the Salina Verde bloom was *Anabaenopsis elenkinii*. This cyanobacterium altered local abiotic parameters such as pH, turbidity, and dissolved oxygen and consequently the overall structure of the microbial community. In Salina Preta, the microbial community had a more structured taxonomic profile. Therefore, the distribution of metabolic functions in Salina Preta community encompassed a large number of taxa, whereas, in Salina Verde, the functional potential was restrained across a specific set of taxa. Distinct signatures in the abundance of genes associated with the cycling of carbon, nitrogen, and sulfur were found. Interestingly, genes linked to arsenic resistance metabolism were present at higher abundance in Salina Verde and they were associated with the cyanobacterial bloom. Collectively, this study advances fundamental knowledge on the composition and genetic potential of microbial communities inhabiting tropical soda lakes.

## Introduction

Soda lakes are characterized by elevated levels of pH and Na-HCO_3_ that result in saline and hypersaline alkaline waters. These ecosystems have been described worldwide, including the East African Rift Valley (Jones et al., 1977; Eugster and Jones, 1979; Ventosa and Arahal, 1989; Duarte et al., 2012), the North and Central Americas (Domagalski et al., 1989; Kazmierczak et al., 2011), Asia (Ma and Edmunds, 2006; Namsaraev et al., 2015), Australia (Hammer, 1986), and Europe (Felföldi et al., 2009; Stenger-Kovács et al., 2014). The extreme environmental conditions of soda lakes (pH above 9 and salinity) are known to be stressful for phytoplankton communities, and however, several microbial and microalgae taxa have adapted to live under these harsh abiotic conditions (Banciu and Muntyan, 2015; Sorokin et al., 2015). It is worth noting that, despite these conditions, moderately saline alkaline lakes are recognized as among the most productive lakes worldwide (Melack and Kilham, 1974; Hammer, 1981; Melack, 1981). Often, seasonal and permanent microbial bloom events are observed in soda lakes (Jones et al., 1998; Grant, 2006). In African environments, blooms have been mostly caused by the dominance of the cyanobacteria genera *Spirulina*–*Cyanospira*–*Arthrospira* (Krienitz et al., 2013), whereas in other soda lakes (e.g., European soda lakes), the dominant taxa have been affiliated to the unicellular genera *Synechococcus–Cyanobium* (Felföldi et al., 2009; Somogyi et al., 2010). Moreover, soda lakes harbor fully functional and diverse microbial communities responsible for the cycling of chemical elements, such as nitrogen (N), sulfur (S), and carbon (C) (Sorokin et al., 2014). However, relatively little is known about the bacterial community composition and genetic potential in these systems, particularly with respect to how these properties change in the context of cyanobacterial blooms (Antony et al., 2013). In the last decade, studies in soda lakes have emphasized the importance of investigating the linkage between the biogeochemical processes and microbial community diversity in order to address the dynamics of these systems (Antony et al., 2013; Sorokin et al., 2015).

Brazilian soda lakes have high alkalinity and pH, similarly to Soda Lakes in East Africa (Ventosa and Arahal, 1989; Duarte et al., 2012) and Hungarian soda pans (Szabó et al., 2017). The alkalinity is a result of the shift in the CO_2_/HCO_3_^-^/CO_3_^2-^ equilibrium, and, due to the absence of Ca^2+^ or Mg^2+^, the CO_3_^2-^ remains soluble at elevated concentrations. In addition, these lakes are saline due to the high concentration of Na-HCO_3_ (Furian et al., 2013). The Pantanal region has a sub-region named Nhecolândia, which has a series of hundreds of soda lakes coexisting with fresh water, in an area of 24,000 km^2^. These are often shallow and small lakes surrounded by the ground soil elevation that forms a barrier to exchanging water/flooding and promotes physical isolation. The occurrence of natural cyanobacterial blooms in Brazilian soda lakes is also common (De-Lamonica-Freire and Heckman, 1996; Oliveira and Calheiros, 2000; Medina-Júnior and Rietzler, 2005). Recent studies have shown that some lake systems have a constant bloom history, whereas others have seasonal blooms or no record of the phenomena. In the case of bloom events, the dominant cyanobacteria species are often affiliated to *Anabaenopsis elenkinii* and *Arthrospira platensis* (Santos and Sant’anna, 2010; Costa et al., 2016). However, a few culture-based studies on these systems have reported the occurrence of other cyanobacteria, including the description of new genera and species (Vaz et al., 2015).

Brazilian soda lakes in the Nhecolândia region are mainly filled with freshwater during the seasonal rainfall cycle, with the largest proportion of rain occurring between October and April (Tarifa, 1986). In addition, some lakes can also be fed by groundwater and surface water during the period of the Taquari river overflow (Furian et al., 2013). In these lakes, the water column level varies by the process of evaporation resulting in the concentration of solutes. Particularly, soda lakes of this sub-region have been studied with respect to the hydrological functioning and inorganic geochemistry (Barbiero et al., 2007, 2008, 2016; Furquim et al., 2010a,b; Furian et al., 2013). Although several biogeochemical functions have been described in Brazilian lakes (Martins, 2012), information on the microbial community inhabiting these systems is scarce. As such, we set a specific focus on fostering our understanding of the microbial community composition and functional potential by using metagenomic comparative analyses. We studied two distinct soda lakes from the sub-region Nhecolândia, one with a permanent cyanobacterial bloom (green-water lake) and one with no bloom record (black-water lake).

## Materials and Methods

### Sample Collection and Characteristics of the Soda Lakes

The green-water lake (Salina Verde, 19°28′13′′S; 56°3′22′′W) and the black-water lake (Salina Preta, 19°26′56′′S; 56°7′55′′W) are located at the ‘Centenário’ farm in the Pantanal region (Nhecolândia, Mato Grosso do Sul, Brazil). Salina Verde was named based on the green color of the water (“verde” in Portuguese), due to the presence of a continuous cyanobacterial bloom. A set of characteristics and abiotic variables of both lakes were previously reported (see Andreote et al., 2014). The water sampling was performed in September 2012, during the dry season. For that, water samples were collected in triplicate using sterile bottles of 50 mL at 0.40 m from the lake borders. Samples were collected at two depths (surface and bottom layers: 0.25 m in Salina Verde and 0.35 m in Salina Preta), and at two time-points within the same day (10:00 am and 3:00 pm). The sampling time was determined according to the natural occurrence of oxygen saturation observed in Salina Verde. Samples were named by abbreviations in which the letters mean: V: Salina Verde, P: Salina Preta, M: morning, A: afternoon, S: surface, and B: bottom.

### Measurement of Abiotic Parameters

The measurements of pH, electrical conductivity, temperature, water turbidity, and dissolved oxygen (DO%) were performed *in situ* at a distance of 0.40 m from the lake edges using multi-parameter probes HI9838, HI98703, and HI9148 (Hanna Instruments Inc., United States). Water samples were centrifuged at 3,200 × *g* for 30 min immediately after sampling. Samples were then filtered (0.45 μm cellulose acetate filters) and stored in a cool and dark room for further analyses. The concentrations of nitrate (NO^3-^), ammonium (NH^4+^), phosphate (PO_4_^3-^), sulfate (SO_4_^2-^), the ions Cl^-^, Na^+^, Mg^2+^, Ca^2+^, and the carbonate alkalinity were measured. Anions and cations were analyzed by ion chromatography (Small et al., 1975) and carbonate alkalinity by acid titration (HCl 0.1 N) (Gran, 1950).

### Light Microscopy

Freshwater samples were visualized under optical microscopy for the identification of the dominant cyanobacterium species in Salina Verde. The morphological characterization of the species was performed according to Komárek and Anagnostidis (1989).

### Total DNA Isolation

Water samples were concentrated by centrifugation at 7,000 × *g* for 5 min and kept at -20°C for further analysis. Total DNA was isolated using the MoBio Power Soil DNA isolation kit (MoBio Laboratories, Carlsbad, CA, United States), following the manufacturer’s protocol. The quantification of the extracted DNA samples was performed by comparison to Low DNA mass ladder and Qubit dsDNA HS assay kit (Thermo Fisher Scientific, Carlsbad, CA, United States). The DNA integrity was checked by electrophoresis on agarose gel (1% w/v).

### Shotgun Metagenomics

The metagenomic libraries (2 lakes × 2 depths × 2 sampling times × 3 replicates) were prepared using the Nextera XT DNA Sample Preparation kit (Illumina, Inc., San Diego, CA, United States), according to the manufacturer’s protocol. The metagenomic libraries were sequenced using the Illumina MiSeq platform (Illumina) and the MiSeq Reagent Kit V3 (600 cycles) (Illumina).

### Comparative Metagenomic Analyses

For the shotgun metagenomic dataset, overlapping reads were paired using PEAR 0.9.5 (Zhang et al., 2014) and filtered for quality with Phred > 30, and a length longer than 80 nt using Seqyclean 1.9.8^1^. The reads were annotated by BLASTX against the NCBI-nr database (November 2016) using DIAMOND (Buchfink et al., 2014). Retrieved annotations were later analyzed using MEGAN6 (Huson et al., 2016). The data presented in the manuscript was categorized using the SEED database for functional assignment (Overbeek, 2005). We also provided an overview of the taxonomic distribution of bacteria across the samples by retrieving bacterial 16S rRNA gene sequences that matched the Silva SSU database. For the taxonomic assignation of reads associated with the distinct biogeochemical processes, we used the FOCUS tool (Silva et al., 2014) with default parameters.

Prior to comparative metagenomic analyses, samples were normalized by taking into account the smallest number of reads of any of the selected samples. To test for statistical differences of SEED annotations across samples, we used the software package STAMP (Parks et al., 2014). The *p*-values were calculated using the two-sided Welch’s *t*-test followed by Benjamini-Hochberg FDR correction (Haynes, 2013).

The biogeochemical pathways of C, N, and S were annotated and mapped using the read counts from the SEED annotations and marker genes (Supplementary Table 1). The heatmap was constructed based on *z*-score transformed functional annotations to improve normality and homogeneity of the variances. Predicted functional annotations that segregated significantly between sample types were identified using random forest analysis (Breiman, 2001) with 1,000 trees followed by the Boruta algorithm for feature selection (average *z*-scores of 1,000 runs > 4) (Kursa and Rudnicki, 2010).

## Results

### Differences in Abiotic Parameters between Soda Lakes

We found several significant differences in the abiotic parameters between both sampled soda lakes (see **Table 1** for details). Taking into account that the lakes were sampled during the dry season, that is, when the water level was low (sampling site Salina Verde 0.25 m depth and Salina Preta 0.35 m), the water temperature and conductivity increased along the sampling day in both lakes. An increase in water turbidity was observed in Salina Verde in the afternoon (**Table 1**), likely due to the natural oxygen saturation observed in this lake around 1 h pm, which causes water bubbling in this particular lake. Alkalinity, pH, and conductivity parameters were found to be relatively higher in Salina Verde than Salina Preta (see **Table 1** for details).

**Table 1:** Physicochemical data of Salina Verde and Salina Preta lakes measured at morning and afternoon (September, 2012).

	Salina Verde			Salina Preta		
	Morning		Afternoon	Morning		Afternoon
Depth (m)		0.25			0.35	
Temperature (°C)	26.8		34.0	28.7		32.7
Conductivity (μS⋅cm^-1^)	2,528		2,741	1,465		1,587
Alkalinity (meq_c_)		22.5			10.6	
pH	9.6		9.5	9.0		8.8
DO (%)^a^	172		327	86		104
Turbidity (NTU)	852		882	90		90
NO_3_^-^ (mmoL⋅L^-1^)		0.012			0.006	
SO_4_^2-^ (mmoL⋅L^-1^)		2.02			2.33	
PO_4_^3-^ (mmoL⋅L^-1^)		0.004			0.005	
NH_4_^+^ (mmoL⋅L^-1^)		0.6			0.001	
Na^+^ (mmoL⋅L^-1^)		18.3			11.4	
K^+^ (mmoL⋅L^-1^)		8.24			4.39	
Ca^2+^ (mmoL⋅L^-1^)		0.35			0.36	
Mg^2+^ (mmoL⋅L^-1^)		0.15			0.21	
Cl^-^ (mmoL⋅L^-1^)		1.64			1.49	

^a^*DO% in Salina Verde varied from 5% at night to 600% during the day (Barbiero et al., 2017)*.

### Taxonomic Composition of Bacterial Communities in Soda Lakes

**FIGURE 1:**
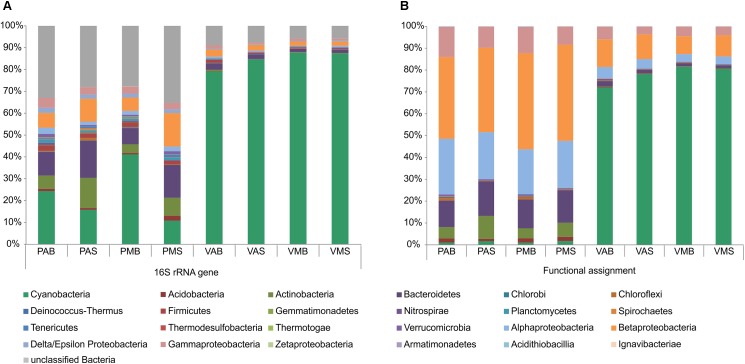
Relative abundances of the main bacterial phyla/classes in Salina Preta and Salina Verde lakes. Data are shown based on bacterial 16S rRNA gene sequences retrieved from the metagenomes **(A)** and functional metagenome annotations **(B)** (see section “Materials and Methods” for details). V: Salina Verde, P: Salina Preta, M: morning, A: afternoon, S: surface, B: bottom.

Information on the taxonomic community composition was obtained by assigning taxonomy to the annotated metagenomic reads. Bacteria were numerically dominant in all the samples (94.56–99.39%), with a minor representation of archaeal sequences (0.06–2.37%), eukaryotes (0.37–2.49%), and viruses (0.11–1.52%). It is worth mentioning that, overall, the taxonomic information carried out by retrieving the bacterial 16S rRNA gene sequences from the metagenomes revealed a roughly similar pattern in community composition as those obtained by annotation of metagenomic reads (see **Table 2** for details). The observed differences were mostly related to variations in a few phyla and classes. Particularly for the annotation of bacterial 16S rRNA gene sequences, a high number of unclassified sequences were obtained for both lakes (minimum of 6.2% in VMB to a maximum of 54.5% in PMS) (**Figure 1**).

**Table 2:** Overall number of metagenomic reads obtained for Salina Verde and Salina Preta samples.

	PAB	PAS	PMB	PMS	VAB	VAS	VMB	VMS
Total nucleotide (Mb)	373,382	534,239	352,370	173,429	178,547	323,728	362,425	286,395
Average read length (bp)	202	214	212	219	196	189	163	167
Total number of reads	1,921878	2,635106	1,828545	808,867	952,827	1,801275	2,248701	1,753792
Taxonomic assigned reads (%)	28.4	33.2	30.1	38.0	47.6	50.4	46.9	39.7
Bacteria (%)	96.09	97.14	94.56	96.56	99.24	99.39	99.26	99.31
Archaea (%)	2.27	0.22	2.37	2.27	0.25	0.06	0.24	0.08
Eukaryota (%)	1.06	1.13	2.49	1.06	0.37	0.39	0.40	0.49
Viruses (%)	0.58	1.52	0.58	0.58	0.15	0.15	0.11	0.12
Functionally assigned reads (%)	37.05	43.54	38.12	52.35	44.52	44.16	39.44	39.24
16S rRNA genes in the metagenome	1477	2746	1947	754	1695	3068	3775	3139
Bacteria 16S rRNA (%)	98.9	100	99.28	100	100	100	99.9	100
Archaea 16S rRNA (%)	1.1	0	0.72	0	0	0	0.10	0

*V: Salina Verde, P: Salina Preta, M: morning, A: afternoon, S: surface, and B: bottom*.

The dominant phylum in Salina Verde was Cyanobacteria (79%) followed by Betaproteobacteria (10%), Gammaproteobacteria (4%), Alphaproteobacteria (4%), and Bacteroidetes (1.6%) (**Figure 1**). The remaining phyla had relative abundances below 1%. The archaeal community was limited to Euryarchaeota, which encompasses methanogenic organisms. The main cyanobacterial genus accounting for the bloom event was affiliated to the nitrogen-fixing *Anabaenopsis* (Nostocales). Optical microscopy of the bloom samples identified the heterocystous filamentous *A. elenkinii* as the dominant planktonic cyanobacterial species. In Salina Verde, the taxonomic composition and the relative abundances of bacterial phyla did not vary between the surface and bottom samples. However, significant differences were only observed in the relative abundances of the Orders Burkoholderiales (*p* = 0.014), Legionellales (*p* = 0.049), Gemmatimonadales (*p* = 7.46e^-3^) and Solibacterales (*p* = 0.014), all having a higher relative abundance in the first sampling time (data not shown).

In Salina Preta, the most abundant bacterial phyla were Betaproteobacteria (40.5%), Alphaproteobacteria (22.1%), Bacteroidetes (14.2%), Gammaproteobacteria (10.8%), Actinobacteria (7.3%), Acidobacteria (1.6%), and Cyanobacteria (1.4%). The remaining phyla had relative abundances below 1%. Most of the reads affiliated to Archaea were assigned to the ammonia-oxidizing organisms of the Order Thaumarchaeota. Also, members of Euryarchaeota were present at low abundances. The samples from the bottom of this lake had a high relative abundance of the green filamentous phototrophs Chloroflexi (*p* = 2.42e^-3^). In addition, the archaeal phylum Thaumarchaeota (*p* = 2.42e^-3^), and the bacterial phyla Nitrospirae (*p* = 0.041), Ignavibacteriae (*p* = 3.45e^-3^), Armatimonadetes (*p* = 0.011), and Spirochaetes (*p* = 0.018) were found to be higher in relative abundances in the bottom samples comparatively to the surface samples (**Figure 1**). No significant difference in taxonomic composition between sampling time was observed in this lake.

### Functional Genetic Potential of Microbial Communities in Soda Lakes

**FIGURE 2:**
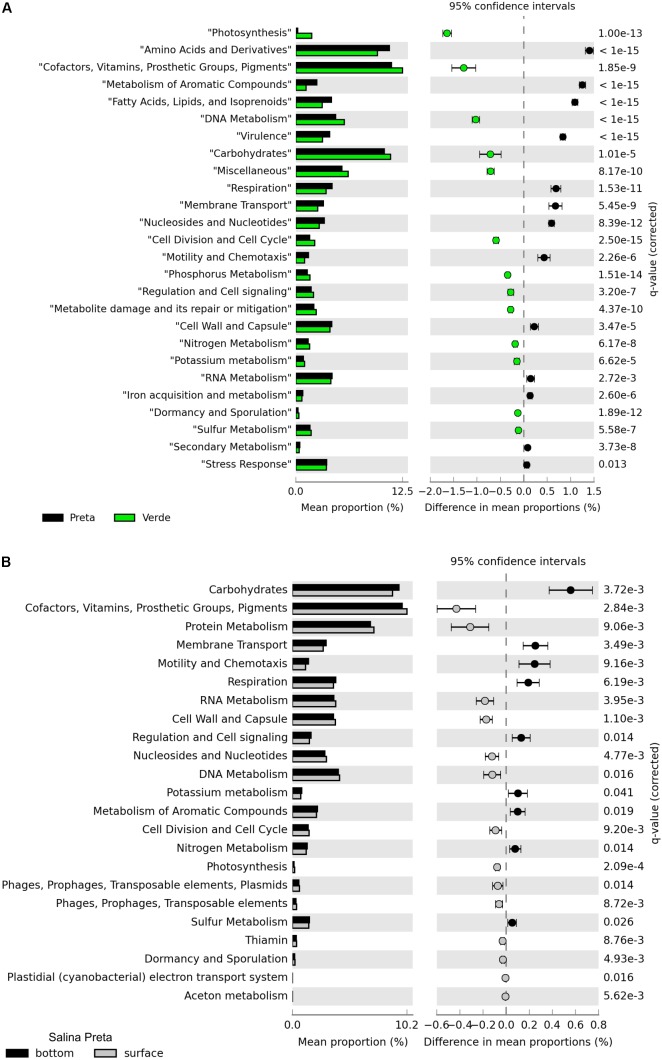
Functional distribution of the metagenomes annotated using the SEED subsystems. Statistically significant differences were found between Salina Preta and Salina Verde lakes **(A)**, and between the bottom and surface water layers in Salina Preta **(B)** (see section “Results” for details).

A total of 13.9 million reads, with average length of 195 bp, totalizing 2,584,515 Mb, were generated in our metagenomic dataset (**Table 2**). The percentage of functionally annotated reads varied from 39.24 to 52.35%, based on the SEED database. Relative abundances of the detected genes were used to infer about the potential relevance of the metabolisms, and to compare across the different sample types. The functional profiles of the metagenomes were compared using STAMP, and significant variations in the genetic potential of biogeochemical cycling processes were found between the lakes with and without cyanobacterial blooms. In addition, significant differences were also found between the bottom and surface samples in Salina Preta (**Figure 2**). No significant difference was observed between the bottom and surface samples in Salina Verde, neither for the two sampling times in both lakes.

**FIGURE 3:**
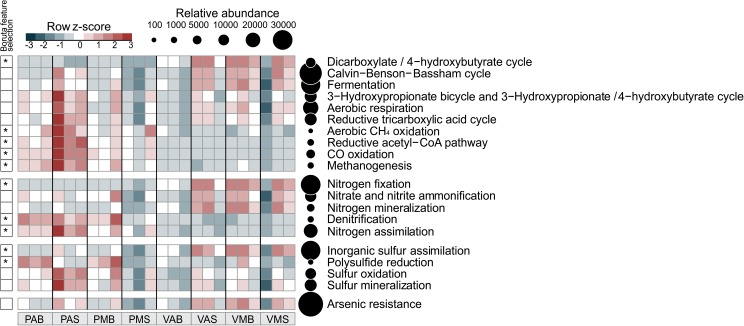
Distribution of functional annotations involved in the C, N, S, and As cycles. The heatmap displays the relative abundance (row *z*-scores) of functional annotations across all samples (triplicate per site). ^∗^Functional annotations that differentially segregated across sites were identified by random forest analysis with Boruta feature selection (average *z*-scores of 1,000 runs > 4). Circles are proportional to the relative abundance of each functional annotation in all samples. V: Salina Verde, P: Salina Preta, M: morning, A: afternoon, S: surface, and B: bottom.

**FIGURE 4:**
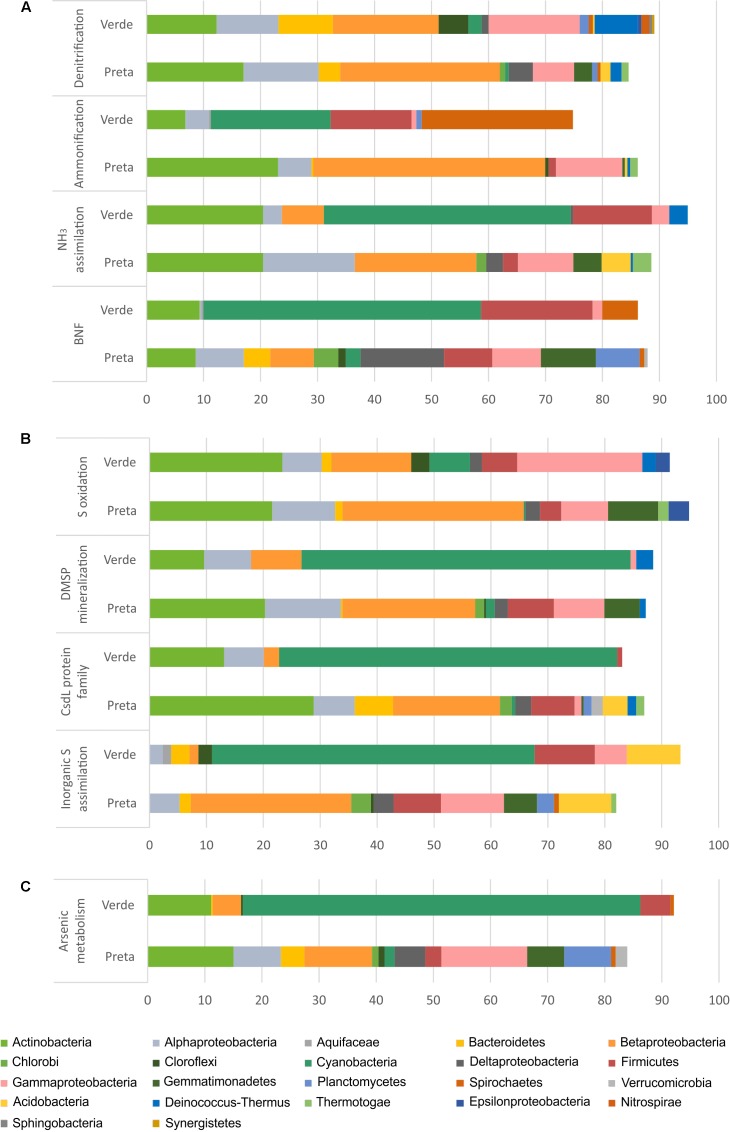
Relative abundances (%) of the main bacterial phyla/classes in Salina Preta and Salina Verde lakes linked to Nitrogen **(A)**, Sulfur **(B)**, and Arsenic **(C)** metabolisms. Data obtained using the SEED genomic approach and the FOCUS tool. This information at order level can be seen in Supplementary Table 2.

As Bacteria and Archaea were the most representative groups of organisms obtained by metagenomics in these lake samples, we focused on them to evaluate the metabolism of N, S, C, and arsenic (As) (**Figure 3**). We set a focus on describing differences across systems (i.e., between lake types), since no strong differences were observed for sampling depth (except for N mineralization in Salina Preta) and sampling time, within each lake system. Interestingly, genes related to the As resistance metabolism showed to vary between the two studied lakes, so data concerning this element was also presented (**Figure 3**). The distribution of bacteria phyla/classes responsible by N, S, and As metabolisms in the two lakes was determined (**Figure 4**). Detailed information of bacterial groups at the taxonomic level of order and genus are presented in Supplementary Table 2.

For the N pathway (Supplementary Figure 1), ammonia assimilation was the most abundant metabolism and higher in Salina Preta than in Salina Verde (**Figure 3**). In Salina Preta, the abundant orders affiliated to this metabolism were Acidimicrobiales (15%), Burkholderiales (11%) and Rhizobiales (10%) (Supplementary Table 2). In Salina Verde, the sequences assigned to this metabolism were affiliated to Cyanobacteria (45%), encompassing mostly *Anabaenopsis* (19%) and *Arthrospira* (13%), followed by sequences affiliated to Actinomycetales (18%) and Thermoanaerobacterales (11%) (**Figure 4** and Supplementary Table 2).

The pathway for biological nitrogen fixation (BNF) was found to be abundant in both metagenomes, being relatively higher in Salina Verde (**Figure 3**), with the dominance of Cyanobacteria (**Figure 4**), represented mainly by the diazotrophic genus *Anabaenopsis* (encompassing a total of 45% of the BNF affiliated sequences) (Supplementary Table 2). The remaining less abundant diazotrophs were assigned to Thermoanaerobacterales, Actinomycetales, and Spirochaetales (encompassing ca. 30% of the BNF-affiliated sequences) (Supplementary Table 2). In Salina Preta, BNF was mostly affiliated with the members of the orders Gemmatimonadales (ca. 9%), Myxococcales, Syntrophobacterales, and Bacillales (ca. 7% each) (Supplementary Table 2). Moreover, considering N supplies, the potential for allantoin utilization and cyanate hydrolysis were also detected in both lakes (Supplementary Figure 1).

The nucleotide sequences encoding nitrate and nitrite ammonification (N mineralization) in Salina Verde were mostly affiliated to orders Spirochaetales (ca. 25%), Chroococcales, and Nostocales (Cyanobacteria) (∼20%), in addition to the Archaea domain (ca. 15%) (Supplementary Table 2). No sequences of the genera *Anabaenopsis* and *Arthrospira* were found associated with the ammonification process. In Salina Preta, this process was mostly affiliated to Burkholderiales (ca. 25%) and Bifidobacteriales (ca. 15%) (Supplementary Table 2). In addition, a relatively higher genetic potential for nitrate and nitrite ammonification was observed in the bottom of this lake (Supplementary Figure 1). Some minor abundant taxa, such as Coriobacteriales, Desulfomonadales, and Myxococcales, were found exclusively at the bottom samples from this lake (Supplementary Table 2).

The genetic potential for denitrification was low in both lakes, being slightly higher in Salina Preta (**Figure 3**). Most of the reads were affiliated to Burkholderiales (ca. 16%) in Salina Preta and Actinomycetales (ca. 15%) in Salina Verde (Supplementary Table 2). Additionally, sequences retrieved from the bottom samples of both lakes and associated with denitrification were affiliated to the domain Archaea (ca. 5–11%).

For the S pathway (Supplementary Figure 2), the highest percentage of the reads was annotated encoding inorganic S assimilation and CsdL protein family (S acceptors), and both were higher in terms of relative abundances in Salina Verde comparatively to Salina Preta (**Figure 3**). In Salina Verde, the reads related to these metabolisms (ca. 40 and 36%, respectively) were affiliated to the bloom-forming *Anabaenopsis* (Supplementary Table 2). Also, 8% of the reads affiliated to inorganic S assimilation and 19% of the reads affiliated to CsdL protein family were assigned to the genus *Arthrospira*. In Salina Preta, these metabolisms were affiliated to a set of different bacterial taxa (**Figure 4**). In addition, archaeal members performing inorganic S assimilation were found exclusively in this lake (ca. 5%). Moreover, the cellular transporters of taurine, glutathione, and alkanesulfonate were found in both lakes (Supplementary Figure 2), suggesting these molecules as additional S sources in these lakes. The dimethylsulfoniopropionate (DMSP) mineralization (demethylation pathway) was mostly affiliated with Cyanobacteria members in Salina Verde (>50%) (**Figure 4**), with reads affiliated to *Anabaenopsis* and *Arthrospira*. In Salina Preta, this metabolism was affiliated to distinct bacterial taxa, with Burkholderiales being the most abundant (44%) (Supplementary Table 2).

The S oxidation pathway (i.e., the multi-enzyme Sox pathway) was mapped in both lakes and sequences were affiliated to a particular set of taxa (**Figures 3**, **4**). In brief, in Salina Verde, sequences were mostly affiliated with Actinomycetales (18%), whereas in Salina Preta, sequences affiliated with Burkholderiales (29%) (Supplementary Table 2). In addition, the Rhodospirillales members were abundant across all the samples (ca. 4.7–10.2%) and assigned as potentially important taxa involved in S oxidation in these systems (Supplementary Table 2).

The six microbial carbon dioxide-fixation pathways were found in the both lakes (**Figure 3**), as follows: the Calvin-Benson-Bassham cycle, the reductive tricarboxylic acid cycle (major catabolic pathway in aerobic organisms); reductive acetyl CoA pathway [in anaerobic bacteria (acetogens) and archaea (methanogens)]; 3-hydroxypropionate-bicycle (in green non-sulfur bacteria); and the archaean exclusives 3-hydroxypropionate/4-hydroxybutyrate and dicarboxylate/4-hydroxybutyrate cycles. Furthermore, the fermentative, methanogenic, and methanotrophic processes were also mapped across the lake samples (see **Figure 3** for details).

Worth noticing, the genetic potential for As resistance was also found in the metagenomes (total of 8,160 reads) and tends to be higher in Salina Verde than in Salina Preta (**Figure 3**). The genes encoding arsenate reductase (ArsC), cellular extrusion (ArsA, ArsB, and ACR3), and As resistance (ArsD and ArsH) were found in both lakes. Both bacteria and archaea members were found as potential As metabolizers (Salina Verde: 98% bacteria and 2% archaea; Salina Preta: 93% bacteria and 7% archaea). The main phylum affiliated with this resistance mechanism in Salina Verde was Cyanobacteria (**Figure 4**), represented by the genus *Anabaenopsis* (ca. 50%), followed by Actinomycetales (11%), Burkholderiales, Clostridiales and Chroococcales (total of 5%), and Oscillatoriales (ca. 3%) (Supplementary Table 2). In Salina Preta, the number of taxa affiliated to As resistance was relatively higher than in Salina Verde (**Figure 4**), and reads were mostly affiliated to the orders Burkholderiales, Planctomycetales, Pseudomonadales, and Actinomycetales (ca. 8% each) (Supplementary Table 2). Additionally, genes encoding dissimilatory As metabolism were poorly mapped in the metagenomes (less than 30 reads related to arsenite oxidase; *Arx*A gene).

## Discussion

In the present study, two soda lakes, i.e., Salina Preta (black-water lake) and Salina Verde (green-water lake with continuous cyanobacterial blooms), were investigated in terms of microbial community structure and the genetic potential for distinct biogeochemical cycles. It is worth mentioning that no direct measurement of salinity was carried out at the sampling time. However, previous studies in these systems describe them as being sub-saline/brackish or hyposaline lakes (Medina-Júnior and Rietzler, 2005; Furian et al., 2013). In line with these arguments, Vavourakis et al. (2016) showed that bacterial taxa often dominate lakes with low salinity values, while archaea are more abundantly present in hypersaline lakes (>250 g.L^-1^). In addition, the taxonomic stratification of one of our lake systems (i.e., Salina Preta) roughly resembles the bacterial community composition previously described in sub-saline Hungarian soda pans (Szabó et al., 2017).

The cyanobacterial bloom observed in Salina Verde was associated with the proliferation of the species *A. elenkinii*. This data corroborated previous studies showing the dominance of *A. elenkinii* in certain Pantanal lakes (Santos and Sant’anna, 2010; Costa et al., 2016). The genus *Anabaenopsis* comprises planktonic species with known distribution in soda lakes, particularly those located in tropical regions (Ballot et al., 2005, 2008; Komárek, 2005). Besides the autotrophic lifestyle, the genus *Anabaenopsis* is also capable of BNF, a feature that confers an adaptive advantage in these systems. The phylum Cyanobacteria seems to play a major role in the geochemistry of Pantanal lakes, but determining the cause(s) of persistent cyanobacterial blooms in hypersaline waters is a daunting task. The rate of photosynthesis that occurs during cyanobacterial blooms results in a sharp increase of pH, as a result of the consumption of CO_2_ and HCO_3_^-^, and the consequent increase of CO_3_^2-^ (Schneider and Le Campion-Alsumard, 1999; Almeida et al., 2011). This increase in pH enhances nutrient release from the sediments at the bottom of these lakes, thus having an influence on the biogeochemical cycling of particular elements. The sum of these factors was previously reported as beneficial for the continuously enhanced proliferation of cyanobacteria in shallow water systems (Gao et al., 2012). Not surprisingly, in our comparative analysis, both pH and alkalinity were higher in Salina Verde (*Anabaenopsis* bloom) than in Salina Preta.

Despite the dominance of *Anabaenopsis* in Salina Verde, the *in silico* reconstruction of the metabolic pathways of N and S cycles (i.e., ammonia assimilation and inorganic S assimilation, both of which supply N and S for biosynthesis) was partially attributed to the genus *Arthrospira*, suggesting that this genus coexists with *Anabaenopsis* in this particular lake system. Corroborating this finding, the recovery of partial 16S rRNA gene sequences from the metagenomes revealed sequences affiliated to *Arthrospira*. In addition, typical *Arthrospira* spiral-shaped filaments were visualized in the water samples from Salina Verde examined under optical microscopy. Collectively, our findings align to other studies that investigated other green-water lakes (named Salina do Meio), by showing the presence of the genera *Arthrospira* and *Anabaenopsis* during bloom events (Santos and Sant’anna, 2010); a fact that has also been previously reported in African soda lakes (Ballot et al., 2008).

The Salina Preta lake (black-water) had a relatively constant DO (ca. 7 mg⋅L^-1^) and turbidity around 90 NTU. The water turbidity is a result of the presence of clay and an elevated amount of dissolved organic matter in the water. Salina Verde is known to have green-water due to the bloom presence; however, the green lakes also present black waters in the absence of bloom events. However, the bloom causes an increase in turbidity and leads to fluctuating values of DO. In this particular lake, the oxygen concentration gradually increases throughout the day, as a result of oxygenic photosynthesis. At night, there is a decrease in oxygen concentration resulting from microbial respiration, which alters local abiotic conditions and consequently affects the microbial community.

The dominance of *Anabaenopsis* in Salina Verde was related to the detection of a higher abundance of genes linked to the metabolism of cell proliferation, DNA replication, and N and S assimilation in the system. Ecologically relevant, this dominance by a single species can be linked to a potential decrease in the community resilience (Shade et al., 2012), since the genetic potential for the metabolism of C, N, and S cycling are restrained across a limited number of taxa, with most of them attributed solely to *Anabaenopsis.* Interestingly, we did not find differences in terms of community composition between the bottom and surface layers of Salina Verde. This could be attributed to the mixing of *Anabaenopsis* cells through the water column caused by the oxygen saturation levels. Comparatively, Salina Preta had a more stratified water column and thus the microbial community differed between the surface and bottom layers, even being a relatively shallow lake.

Nitrogen has been described as a limiting nutrient in Brazilian soda lakes, particularly due to the low ratio of nitrogen/phosphorous, which reduces the bioavailability of nitrogenous compounds in these systems (Mourão, 1989). In this study, the presence of a cyanobacterial bloom was related to an elevated detection of BNF potential in Salina Verde. In Salina Preta, despite the mechanisms involved in BNF in soda lakes under oxic conditions are unknown, several bacterial taxa were affiliated to this process. The total N content in these two lakes was previously reported, revealing a 10-fold higher value in Salina Verde (Machado, 2016) than in Salina Preta. The *Anabaenopsis* bloom certainly contributes to this input of N into Salina Verde via BNF. In addition, the N bioavailability by mineralization, and the potential metabolism for allantoin utilization (N source in anaerobic conditions) and cyanate hydrolysis (N source in alkaline conditions) may likely contribute to the N supply in these lakes.

The genes involved in the conversion of ammonia to nitrate and nitrite (i.e., nitrification) were not detected in the metagenomes. However, the genetic potential for ammonia, nitrite, and nitrate assimilation processes was abundantly found in both lakes. It is known that nitrification can be low or absent at high salt concentrations (Sorokin, 1998) and that saline lakes often have an external source of NO_2_^-^ and NO_3_^-^ (Oren, 2001). We also found that the N loss by denitrification was poorly mapped in these systems, since denitrification depends on anoxic local conditions, and as such, is more likely to be performed by particular taxa that occur in the bottom sediments of these lakes.

The genetic potential of S metabolism was found in both lakes, with a large proportion of genes involved in the S oxidation. The Sox pathway, a key mechanism allowing the oxidation of thiosulfate in sulfate was mapped in the metagenomes. Sulfide and thiosulfate are reduced inorganic S molecules normally abundant in the environment and the conversion of these molecules in the bioavailable sulfate is mainly performed by bacterial action, during the oxidative part of the S cycle (Friedrich et al., 2005). In these lakes, this process was driven by chemolithoautotrophic haloalkaliphilic sulfur-oxidizing bacteria members of the orders Actinomycetales, Burkholderiales, and Rhodospirillales. We also found a few genes involved in polysulfide reduction, a process more prone to occur in the sediments rather than in the water of these lakes. Corroborating this argument, the S cycle has been previously suggested to be one of the most active metabolisms in soda lakes (Sorokin et al., 2011), where the metabolism of S reduction takes place in the anoxic sediment layers (Sorokin et al., 2014).

In terms of the C cycling, soda lakes rank among the most productive natural ecosystems in the world, being active on atmospheric C sequestration, but also contributing significantly for the production and emission of greenhouse gases (GHG) (Antony et al., 2013). It is important noticing that the contribution of tropical green-water lakes (as Salina Verde) and black-water lakes (as Salina Preta) for GHG emissions is different. Green-water lakes are considered as CO_2_ sinks, reaching up to 10 times higher content of dissolved organic carbon (DOC) than black-water lakes, which are considered CO_2_ sources (Machado, 2016; Barbiero et al., 2017). In our studied system, the six pathways of atmospheric CO_2_ fixation (Sato and Atomi, 2010) were mapped, illustrating a diverse microbial composition, including a set of bacterial and archaeal members potentially involved in C sequestration. As expected, the Calvin-Benson-Bassham cycle (performed by photosynthetic bacteria) was highly abundant in Salina Verde, a process directly linked to the presence of a cyanobacterial bloom, which also leads to major rates of CO_2_ fixation in this lake. As such, it is unquestionable that cyanobacterial blooms constitute an important event for the dynamic of C sequestration and bioavailability in these lake systems.

Considering the metabolism of methane, both the processes methanotrophy (aerobic CH_4_ oxidation) and methanogenesis (anaerobic process preferentially active on sediments and performed by specialized archaea taxa) were found in the metagenomes. Both of the investigated lakes are considered as CH_4_ sources, with Salina Verde having a higher rate of CH_4_ emission than Salina Preta (Barbiero et al., 2017). In Salina Verde, the percentage of DO varied from 5 to 600% (night and day, respectively), due to the photosynthetic and respiratory activities. This shift leads to a microaerobic condition that may promote methanogenesis during the night, which also results in water bubbling caused by the oxygen saturation, thus favoring CH_4_ emission during the day. Despite these lines of evidence, it is also important to consider that (i) methanotrophs are also responsible for a significant consumption of CH_4_ in saline and alkaline lakes (Joye et al., 1999; Khmelenina et al., 2000), and (ii) methanogenesis is a process more likely to occur at elevated rates in the sediments of these lakes.

The waters of the studied lakes have high As content, with values ranging from 0.05 to 0.5 mg⋅L^-1^, mostly found in the form of arsenate – As(V), and the As content being higher in Salina Verde than Salina Preta (Barbiero et al., 2007). The transformation of As(V) (lower toxicity/lower bioavailable) to As(III) (higher toxicity/higher bioavailable) is favored by alkaline conditions, occurring through two main mechanisms: (i) the resistance mechanism (As detoxification efflux system), and (ii) the dissimilatory arsenate reduction, which is predominant under anoxic conditions (Oremland et al., 2004; Kulp et al., 2006; Das et al., 2016). The mechanism of dissimilatory arsenate reduction was only poorly mapped in our metagenomes, likely because this is a metabolism that takes place in the sediment of these lakes. However, we found the evidence for the genetic potential of As resistance in the lake samples, a feature occurring across several microbial taxa, including *Anabaenopsis.* The presence of arsenate detoxification genes has been reported in a variety of pico and unicellular cyanobacteria (López-Maury et al., 2003; Dyhrman and Haley, 2011; Sánchez-Riego et al., 2014), including *Arthrospira* spp. (Lefort et al., 2014; Guo et al., 2016). The dominance of *Anabaenopsis* in Salina Verde and the high number of reads linked to As resistance affiliated to Cyanobacteria suggest this genus as a potentially important As metabolizer in the system.

To the best of our knowledge, this study constitutes the first report that explores microbial community composition and functional potential in Brazilian soda lakes. The metagenomic approach unveiled the relations among distinct microbial taxa and their potential role in the biogeochemical transformations of C, N, and S, in a comparative framework that included two distinct lake systems (Salina Verde and Salina Preta). Our data provided valuable insights into the metabolism of important inorganic compounds taking place in these ‘extreme’ environments. Future efforts should be made in investigating the taxonomic and functional profiles of a broader range of soda lakes, with and without cyanobacterial blooms, including their seasonal variations and lake vertical profile stratification. In addition, sediments of these lakes should be taken into consideration to assess important anoxic metabolic processes. Thus, the present study collectively contributes to a more comprehensive overview of the biogeochemical cycling mediated by microbial communities in these systems.

## Nucleotide Sequence Accession Number

The sequence data (total of 24 metagenomes) have been deposited in the MG-RAST database under the project named Pantanal and accession numbers: mgm4575354.3–mgm4575377.3.

## Author Contributions

MF conceived the study. LB and AR-F collected the samples. GM and JR performed the experiments. AA, JR, GM, FD-A, and BS analyzed the data. All authors were involved in writing the paper and had final approval of the manuscript.

## Conflict of Interest Statement

The authors declare that the research was conducted in the absence of any commercial or financial relationships that could be construed as a potential conflict of interest.

## Footnotes

1 https://bitbucket.org/izhbannikov/seqyclean
