# Supplementary material for: Contrasting the Genetic Patterns of Microbial Communities in Soda Lakes with and without Cyanobacterial Bloom
Source: Front Microbiol. 2018 Feb 22;9:244. doi: 10.3389/fmicb.2018.00244 (PMC5827094; doi:10.3389/fmicb.2018.00244)
Supplement: Supplementary file 1 [file Table_1.docx]

**Supplementary Table S1**. Marker genes used in the present work to evaluate the metabolism of C, N, S and As.

| **Cycle** | **Step** | **Origin** |
| --- | --- | --- |
| **CARBON** | **Calvin-Benson-Bassham cycle** | SEED |
|  | **Reductive tricarboxylic acid cycle** | Isocitrate dehydrogenase |
|  |  | Indolepyruvate ferredoxin oxidoreductase |
|  |  | Phosphoenolpyruvate carboxylase |
|  | **Reductive acetyl-CoA pathway** | Carbon monoxide dehydrogenase |
|  | **3-Hydroxypropionate/malyl-CoA cycle and 3-Hydroxypropionate/4-hydroxybutyrate cycle** | Fumarate reductase flavoprotein |
|  |  | acetyl-CoA carboxylase |
|  |  | Propionyl-CoA carboxylase |
|  | **Dicarboxylate/4-hydroxybutyrate cycle** | Phosphoenolpyruvate carboxylase |
|  |  | Pyruvate oxidoreductase |
|  | **Aerobic CH_4_ oxidation** | Methane/phenol monooxygenase |
|  | **Aerobic respiration** | Cytochrome c oxidase |
|  | **CO oxidation** | Carbon monoxide dehydrogenase |
|  | **Fermentation** | SEED |
|  | **Methanogenesis** | SEED |
| **NITROGEN** | **Nitrate and nitrite ammonification** | SEED subsystem |
|  | **Denitrification** | SEED subsystem |
|  | **Nitrogen assimilation** | assimilatory nitrate reductase |
|  |  | glutamine synthetase |
|  |  | glutamate synthase |
|  | **Nitrogen fixation** | SEED subsystem |
|  | **Nitrogen mineralization** | SEED subsystem |
| **SULFUR** | **Inorganic sulfur assimilation** | SEED subsystem |
|  | **Sulfur DMSP mineralization** | SEED subsystem |
|  | **Sulfur oxidation** | SEED subsystem |
|  | **Polysulfide reduction** | sulfide reductase |
| **ARSENIC** | **Arsenic resistance mechanism** | SEED |
