# Supplementary figures and images for: Contrasting the Genetic Patterns of Microbial Communities in Soda Lakes with and without Cyanobacterial Bloom
Source: Front Microbiol. 2018 Feb 22;9:244. doi: 10.3389/fmicb.2018.00244 (PMC5827094; doi:10.3389/fmicb.2018.00244)

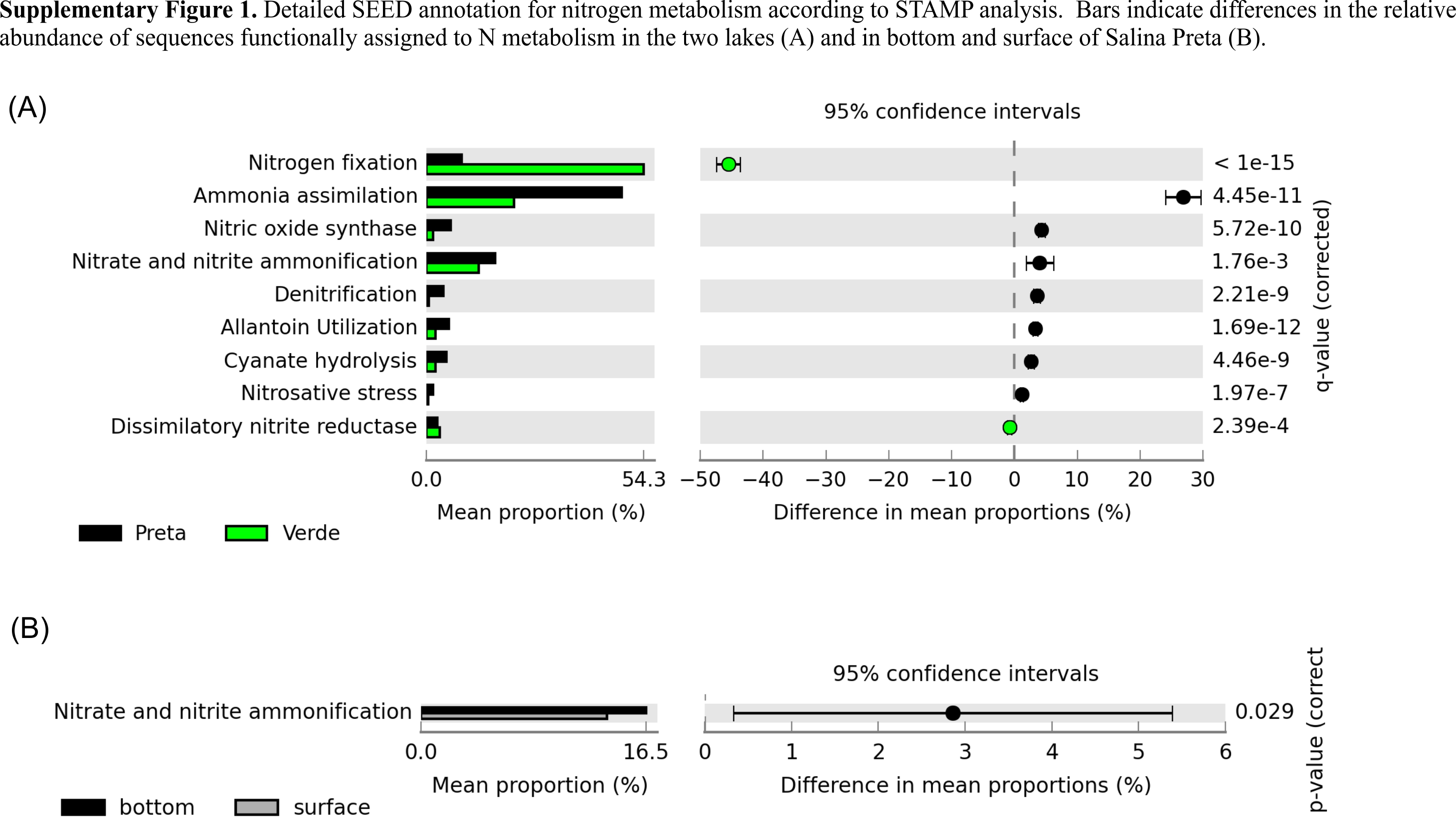

Supplement: Supplementary file 3 [file Image_1.JPEG]

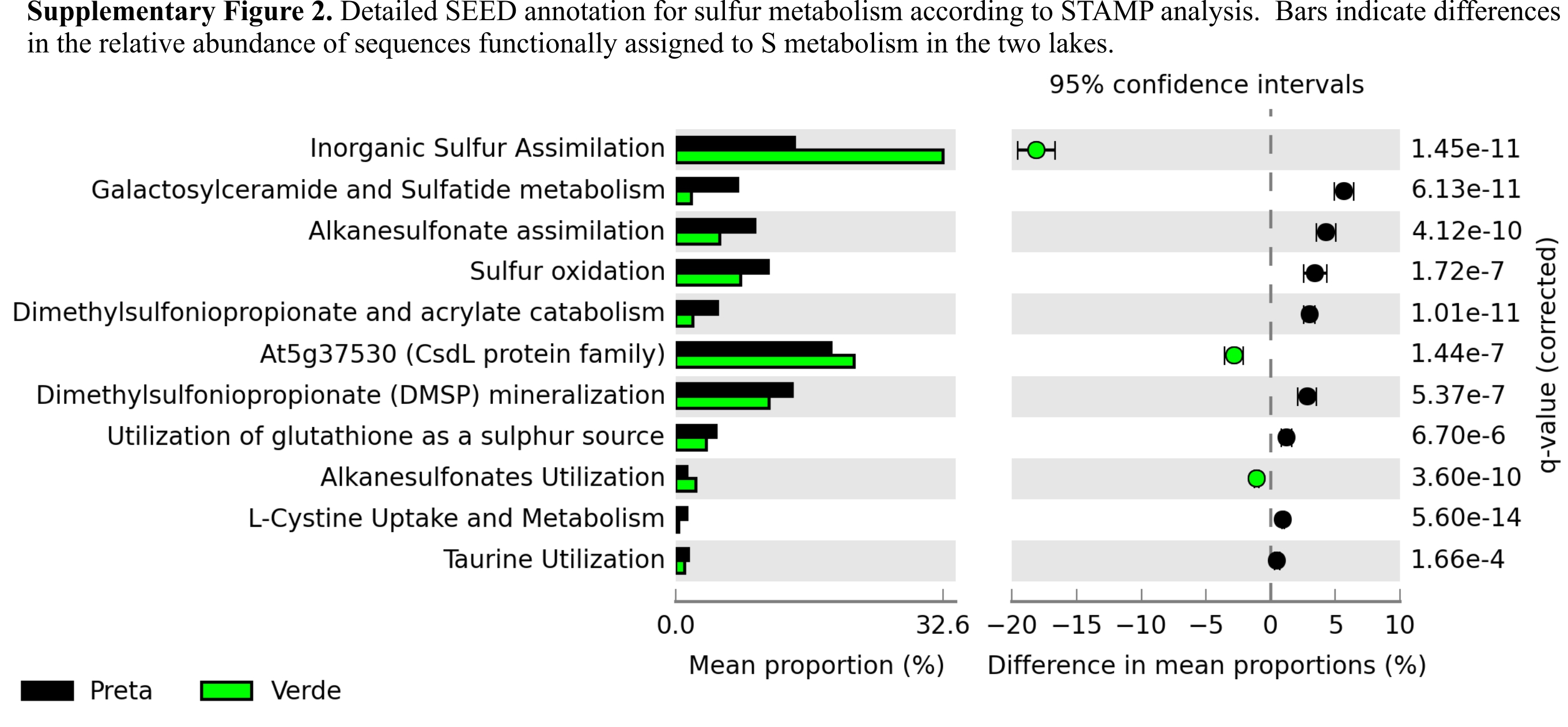

Supplement: Supplementary file 4 [file Image_2.JPEG]
